# Supplementary material for: Musculoskeletal pain among desk-based officials of Bangladesh: Association with mental health and individual factors
Source: PLOS Glob Public Health. 2023 Apr 19;3(4):e0001689. doi: 10.1371/journal.pgph.0001689 (PMC10115271; doi:10.1371/journal.pgph.0001689)
Supplement: S1 File — (DOCX) [file pgph.0001689.s001.docx]

**S1 File:**

**Flow chart of participants inclusion for analysis**

| A total of 615 respondents from the different government and non-government organization were asked to participate in the study |
| --- |

*Respondents participated and*

*were interviwed (n=589)*

| Collected complete questionnaire from 530 participants after receiving consent |
| --- |

*Respondents were excluded*

*due to missing value (n=4)*

| Data from 526 participants were analyzed |
| --- |
