# Supplementary material for: Musculoskeletal pain among desk-based officials of Bangladesh: Association with mental health and individual factors
Source: PLOS Glob Public Health. 2023 Apr 19;3(4):e0001689. doi: 10.1371/journal.pgph.0001689 (PMC10115271; doi:10.1371/journal.pgph.0001689)
Supplement: S2 File — (DOCX) [file pgph.0001689.s002.docx]

**S2 File:**

**Musculoskeletal pain among desk-based officials of Bangladesh: association with mental health and individual factors**

**QUESTIONAIRE**

Serial No……………………………………. Date………………………………….

Address……………………………...

| 1. **Socio-demographic characteristics:** |  |
| --- | --- |
| 1. Date of Birth/ Age | Answer: |
| 1. Gender | 1. Male 2. Female |
| 1. Religion | 1. Muslim 2. Others |
| 1. Height | ________(cm) |
| 1. Weight | ________(kg) |
| 1. Organization type | 1. Government 2. Private 3. Multinational 4. NGO 5. Others |
| 1. Monthly income | ________(BDT) |
| 1. Number of family members? |  |
| 1. Floor living in? | 1. GF- 2^nd^ 2. 3^rd^ -5^th^ 3. 6^th^ floor or upper |
| 1. Lift facility in house? | 1. Yes 2. No |
| 1. **Activity related factors:** |  |
| 1. Total amount of Physical activity/day? | 1. No  2. <60 minutes  3. ≥ 60 minutes |
| 1. Weekly official working hours? | 1. 40 hours or less 2. 41-50 hours 3. More than 50 hours |
| 3. Sedentary activities (Hours of watching TV, Computer or mobile games, reading story book?) | 1. < 2 hours 2. 2-4 hours 3. > 4 hours |
| 1. **Musculoskeletal pain related factors:** |  |
| Pain or discomfort in upper extremity (using the visual analogue scale) | 1. No pain 2. Mild pain 3. Moderate pain 4. Severe |

| 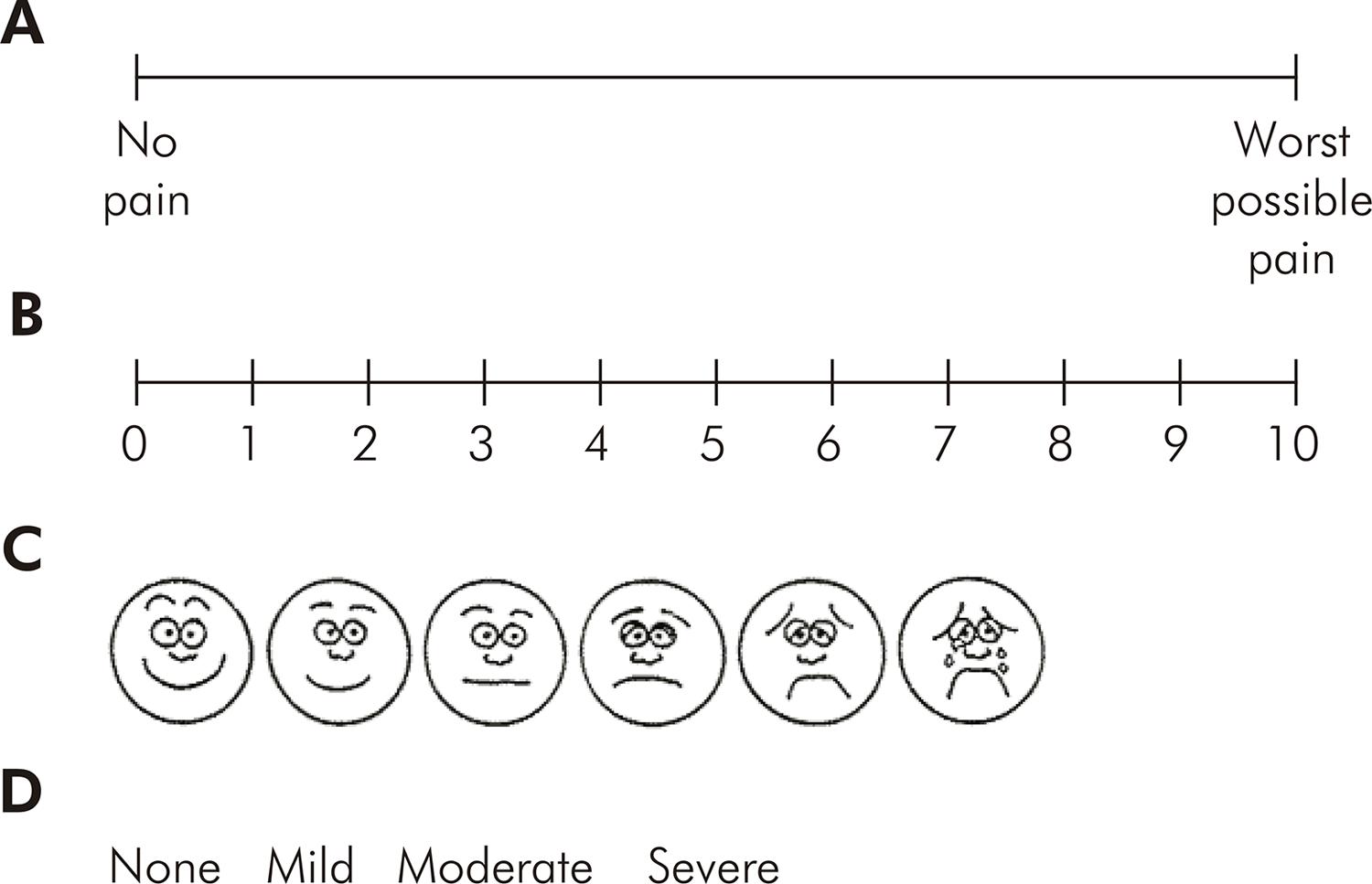 |
| --- |
| **Visual analog scale (VAS)** |

**Hospital Anxiety and Depression Scale (HADS)**

Tick the box beside the reply that is closest to how you have been feeling in the past week.

Don’t take too long over you replies: your immediate is best.

| **D** | **A** |  | **D** | **A** |  |
| --- | --- | --- | --- | --- | --- |
|  |  | **I feel tense or 'wound up':** |  |  | **I feel as if I am slowed down:** |
|  | 3 | Most of the time | 3 |  | Nearly all the time |
|  | 2 | A lot of the time | 2 |  | Very often |
|  | 1 | From time to time, occasionally | 1 |  | Sometimes |
|  | 0 | Not at all | 0 |  | Not at all |
|  |  |  |  |  |  |
|  |  | **I still enjoy the things I used to enjoy:** |  |  | **I get a sort of frightened feeling like 'butterflies' in the stomach:** |
| 0 |  | Definitely as much |  | 0 | Not at all |
| 1 |  | Not quite so much |  | 1 | Occasionally |
| 2 |  | Only a little |  | 2 | Quite Often |
| 3 |  | Hardly at all |  | 3 | Very Often |
|  |  |  |  |  |  |
|  |  | **I get a sort of frightened feeling as if something awful is about to**  **happen:** |  |  | **I have lost interest in my appearance:** |
|  | 3 | Very definitely and quite badly | 3 |  | Definitely |
|  | 2 | Yes, but not too badly | 2 |  | I don't take as much care as I should |
|  | 1 | A little, but it doesn't worry me | 1 |  | I may not take quite as much care |
|  | 0 | Not at all | 0 |  | I take just as much care as ever |
|  |  |  |  |  |  |
|  |  | **I can laugh and see the funny side**  **of things:** |  |  | **I feel restless as I have to be on the**  **move:** |
| 0 |  | As much as I always could |  | 3 | Very much indeed |
| 1 |  | Not quite so much now |  | 2 | Quite a lot |
| 2 |  | Definitely not so much now |  | 1 | Not very much |
| 3 |  | Not at all |  | 0 | Not at all |
|  |  | **Worrying thoughts go through my**  **mind:** |  |  | **I look forward with enjoyment to**  **things:** |
|  | 3 | A great deal of the time | 0 |  | As much as I ever did |
|  | 2 | A lot of the time | 1 |  | Rather less than I used to |
|  | 1 | From time to time, but not too often | 2 |  | Definitely less than I used to |
|  | 0 | Only occasionally | 3 |  | Hardly at all |
|  |  |  |  |  |  |
|  |  | **I feel cheerful:** |  |  | **I get sudden feelings of panic:** |
| 3 |  | Not at all |  | 3 | Very often indeed |
| 2 |  | Not often |  | 2 | Quite often |
| 1 |  | Sometimes |  | 1 | Not very often |
| 0 |  | Most of the time |  | 0 | Not at all |
|  |  |  |  |  |  |
|  |  | **I can sit at ease and feel relaxed:** |  |  | **I can enjoy a good book or radio or TV**  **program:** |
|  | 0 | Definitely | 0 |  | Often |
|  | 1 | Usually | 1 |  | Sometimes |
|  | 2 | Not Often | 2 |  | Not often |
|  | 3 | Not at all | 3 |  | Very seldom |

Please check you have answered all the questions Scoring:

Total score: Depression (D)________ , Anxiety (A) ________

0-7 = Normal

8-10 = Borderline abnormal (borderline case)

11-21 = Abnormal (case)
