# Supplementary material for: Musculoskeletal pain among desk-based officials of Bangladesh: Association with mental health and individual factors
Source: PLOS Glob Public Health. 2023 Apr 19;3(4):e0001689. doi: 10.1371/journal.pgph.0001689 (PMC10115271; doi:10.1371/journal.pgph.0001689)
Supplement: S3 File — (DOCX) [file pgph.0001689.s003.docx]

**S3 File:**

**Descriptive statistics of sociodemographic characteristics of the desk-based officials of Dhaka city (n=526)**

| **Variables** | **Category** | **Frequency** | **Percent** |
| --- | --- | --- | --- |
| Age | < 35 years | 203 | 38.6 |
|  | 35-54 years | 237 | 45.1 |
|  | > 54 years | 86 | 16.3 |
|  | Mean±SD | 39.58±10.56 | |
| Gender | Female | 156 | 29.7 |
|  | Male | 370 | 70.3 |
| BMI | Normal (18.5-24.9 kg/m^2^) | 132 | 25.1 |
|  | Overweight (25.0-29.9 kg/m^2^) | 134 | 25.4 |
|  | Obese (≥30.0 kg/m^2^) | 260 | 49.4 |
|  | Mean±SD | 24.8±2.66 | |
| Monthly income | ≤ 35000 BDT | 156 | 29.7 |
|  | 35000-60000 BDT | 128 | 24.3 |
|  | > 60000 BDT | 242 | 46.0 |
|  | Mean±SD | 74047±57524 | |
| Organization type | Government | 286 | 54.4 |
|  | Private | 240 | 45.6 |
