# Supplementary material for: Musculoskeletal pain among desk-based officials of Bangladesh: Association with mental health and individual factors
Source: PLOS Glob Public Health. 2023 Apr 19;3(4):e0001689. doi: 10.1371/journal.pgph.0001689 (PMC10115271; doi:10.1371/journal.pgph.0001689)
Supplement: Attachment — Submitted filename: Response to Reviewers.pdf [file pgph.0001689.s004.pdf]

Dear Editor,

PLOS Global Public Health

Manuscript title: **Musculoskeletal pain among desk-based officials of Bangladesh: Association with mental health and individual factors**

Thank you for the opportunity to respond to the reviewer's comments. We found the reviewers' comments and feedback very helpful in improving the manuscript. Please find below a point-by-point response to each of the reviewers' comments.

We look forward to hearing from you.

Yours sincerely,

Md Shahjalal (corresponding author)
Public Health Department
North South University, Bangladesh

Reviewer #1

Methods section:

1. There should be clarity about the study population and line 92 mentions "two city corporations," which I think is not enough.

Authors' response: Thank you for your comment. We made it clear in the revised manuscript. Our study was conducted among Bangladeshi desk-based officials. The sample was taken from two conveniently selected city corporations of Dhaka between November 2020 and March 2021. Dhaka is Bangladesh's capital and largest city, with two city corporations, the North and the South. ***Now reads it****- A cross-sectional study was conducted among the desk-based officials in Dhaka, Bangladesh. Dhaka is Bangladesh's capital and largest city, with two city corporations, the North and the South. The sample was collected from conveniently selected these two-city corporations from November 2020 to March 2021. We arranged an interview if there was a desk-based officer of in the household with at least one-year of experience. However, we excluded subjects who took any stress-related medicine for psychiatric symptoms or illegal drugs and refused to give written consent.*

Please see page:4, lines: 94-98

2. Exclusion of participants taking stress-related medicines on line 94 will need to be justified as it is unclear if the medicines are for treatment or prevention of stress (Antidepressants, Anxiolytics, Supplements?)

Authors' response: Thank you. We have clarified it in the revised manuscript.

Now it reads- However, we excluded subjects under psychiatric treatment and refused to give written consent.

Please see page:4, lines: 99-100.

3. The calculated sample size on line 97 was 345; however, 526 responses were collected (line 98), which is more than 150% of the calculated sample size. The researchers may need to explain why improving accuracy may not be tenable.

Authors' response: Thank you. The sample size calculation section has now been revised.

Now reads it- *The sample size was calculated based on the assumptions of an alpha of 0.05, a confidence interval of 95% and a prevalence of musculoskeletal condition of 30.4% in Bangladesh. The sample size thus calculated was 345. After adding a 20% non- response rate the final sample size was calculated to be 414. However, we collected in total of 526 data to represent the population better, avoid sampling error and have more accuracy of the result.*

Please see page: 4, line: 101-105

4. There was no indication that a physical examination was performed on the respondents, but calculations of Body Mass Index (BMI) were made (Line 135). The researchers may need to explain why they took the approach.

Authors' response: Thank you for your comment. In Supplementary file 2, we have attached the questionnaire. The questionnaire indicated that we had collected the data of height in inches and weight in kilograms from the participants. Afterwards, we calculated each participant's BMI. However, we have revised the line.

Now reads it- *Age group (<35 years, 35-54 years and >54 years), gender (male vs female), height (inches), weight (kilogram), monthly income in Bangladeshi Taka (BDT) (≤35000 BDT (about 340 US dollar), 35000-60000 BDT and >60000 BDT), organization (government vs private) were among the socio-demographic characteristics.*

Please see page: 5, lines: 123-124

5. Lines 100 and 101 indicated that the participants completed the questionnaires. Line 101 does not clearly indicate that written informed consent was obtained, as stated in line 126.

Authors' response: Thank you for valid point. Now we have indicated that written consent was obtained from participants. ***Now reads it-*** *However, we excluded subjects who took medicine for psychiatric symptoms or illegal drugs and refused to give written consent.*

Please see page: 4, line:99-100

Results Section

6. There should be clarity on what BDT means (lines 136,137), and maybe an indication of its value compared to an internationally recognized currency would be welcomed.

Authors' response: We have clarified the BDT and compared BDT with USD in the revised manuscript.

Please reads it*- monthly income in Bangladeshi Taka (BDT) (≤35000 BDT (about 340 US dollar), 35000 to 60000 BDT and more than 60000 BDT.*

Page: 5, lines: 124-125

7. Lines 172 to 174 reported finding relating to the presence of Anxiety or Depression among study participants. Were there patients who had BOTH Anxiety and Depression?

Authors' response: Thank you for your valid point. We have showed the percentage of patient who had both anxiety and depression. Please see the revised manuscript.

Please see page: 8, lines: 194-195

Authors' response: Thank you. The figure 2 has been revised based on your suggestion. Please see the revised Figure 2.

Discussion section:

9. I think the discussion needs to be more robust, and not just restate findings in the results section. Lines 192, 205,206 mentioned the word "bothered". I would suggest that the researchers consider the use of another word.

Authors' response: Thank you for your suggestions. The authors firmly agree with you and revised the whole discussion section according to your suggestion. Additionally, we have replaced bothered with irritated in the revised manuscript. Please see the revised discussion.

Please see page: 9-11, lines: 206- 272

10. Researchers may wish to indicate that the Hospital Anxiety and Depression Scale (HADS) is a screening tool, and not used to diagnose either Anxiety or Depression.

Authors' response: The Anxiety and Depression Scale (HADS) scale is not used to diagnose of a mental health disorder. It is only used to identify the presence and severity of symptoms of anxiety and depression in people who are undergoing medical treatment for physical health problems. However, we have used it as a screening tool. Please see the revised manuscript.

Please see page: 6, lines: 135-142

Reviewer #2

1. Authors need to consider more recent references as most studies referenced are dated a while back.
Authors' response: Thank you for your suggestion. The references have now been updated in the revised manuscript.
Please see page:12-17, lines: 219- 411

1. Lines 35 & 117- Replace "analogue" with "analog."
Authors' response: The replace has been done.
Please see revised manuscript.

2. Line 39- Check the spelling of "prevalences" and Line 42- Insert "the" before the house.
Authors' response: We have checked and corrected all spelling.
Please see revised manuscript.

4. Line 46- The study revealed a high prevalence of MS pain and mental health problems among desk-based officials of where? The study area should be stated.
Authors' response: Thank you for your valid point. We have revised the sentence and mentioned the study area. ***Now reads it****- This study has revealed a relatively high prevalence of MS pain and mental health problems among Bangladeshi desk-based officials.*
Please see page: 2, line:49 -50

5. Line 47- "Preventive measures" should replace "preventive measure."
Authors' response: We have replaced it. Please see revised manuscript.

6. Line 73- Change "variable" to "variables." and Line 79- Replace "garments" with "garment."
Authors' response: Done.
Please see revised manuscript.

7. Line 80- Replace "community" with "communities." (Restructure the sentence into an active form)
Authors' response: We have replaced it and reconstructed the sentence into active form.
Now it reads- In recent years, a growing number of studies were conducted in Bangladesh on MS disorders/pain with different study participants and locations, where two studies were conducted among garments workers, and a study was conducted among rural communities.
Please see page: 4, line: 81-83

8. Line 82- Delete "s" in pandemics and Line 91- Insert a hyphen (-) between cross and sectional as well as Line 92- Replace "were" with "was."
Authors' response: Done.
Please see the revised manuscript.

9. Line 99- How were the questionnaires administered? Were they self-administered or interviewer's administered?
Authors' response: Thank you for your comment. We have clarified it in the revised manuscript.
Now reads it*- Data were collected using a validated English version self-administrated questionnaire through face to face interview from study participants.*
Please see page:5, line: 106-107

10. What data analysis software was used, and how were data managed?
Authors' response: Thank you. We have revised the section. *Now it reads- After the collection of data, an MS excel sheet was created. All data were managed in the MS excel sheet; after that, Stata version 14 were used for analytical exploration. Descriptive statistics of demographic characteristics and frequency of exposure to musculoskeletal pain were calculated. In the analytical portion, multinomial logistic regression analysis was used to determine which socio-demographic characteristics (including age, gender, income,*

BMI, organization type, floor living, physical activity/day, weekly working time, sedentary activities, lift facility in house, level of anxiety and level of depression) associated with having MS pain. Adjusted regression analysis was performed based on individual explanatory variables only. In the adjusted final model, only potential explanatory variables were considered if their covariate had a label that showed statistical significance with a p-value of less than 5% in the chi square model.
Please see page:5, lines:109-118

11. Line 94- Why did you limit included year of experience to one year? Any scientific or significant reason? Replace "subject" with "subjects. And " Line 93- Replace "of" with "in." and
Authors' response: Thank you. We have excluded those who had experienced as a desk-based professional less than one year because MS pain takes time to initiate and it is scientifically proved (1).

12. Line 95- Insert "a" before the confidence interval and Line 96- Insert "a" before prevalence.
Authors' response: Done
Please see the revised manuscript.

13. Line 98- Restructure the sentence into an active form and Line 100- Insert "a" before validated.
Authors' response: Done

14. Line 108- Delete "in." and Lines 110 & 112- Replace "3" with "three."
Authors' response: Done.

15. Line 112- Replace "either" with "whether" and insert "a" before lift in their home and Line 116- Replace "extremity" with "extremities."
Authors' response: Done.

16. Line 117- Insert "a" before visual and Line 118- Insert a hyphen (-) between chi and square as well as Lines 119 & 122- Replace "in" with "into."
Authors' response: Done.

16. Line 121- Delete "the" before anxiety and Line 132- Change "consistent" to "consisted." As well as Line 135- Insert "a" before maximum, "of" before 33.6kg/m2, "a" before minimum, and "of" before 18.7kg/m2.
Authors' response: Done.

17. Line 136- Insert "to be" before 3000,000 BDT and "a" before mean and Line 149- Insert "that" before the respondents.
Authors' response: Done.

18. Can the tables be re-formatted, so they only display the adjusted 95%CI rather than the unadjusted and adjusted values? Then, what was reported can be reported in the pros portion of the results section.
Authors' response: Thank you. We have revised the table based on your comments. Please see the revised tables.

19. Results should not be repeated in the discussion section, but observations can be adequately compared to other studies using comparative adjectives.
Authors' response: Thank you for your valid point. We have revised the discussion section extensively based on your suggestion. Please see the revised manuscript
Please see page: 9-11, lines: 206- 272

20. Generally, the entire manuscript should be thoroughly reviewed by a native English speaker. This is to correct all grammar and punctuation mistakes. In this comments section, several errors have been identified, but there are many more that need to be rectified.
Authors' response: Thank you for your comment. We have revised the whole manuscript and corrected grammar and punctuation mistakes accordingly. Please see the revised manuscript.

Thank you for insightful comments.

……………………………………………………………………………….

Reference:
[1]. https://bestpractice.bmj.com/topics/en-us/834
